# Characterization of extracts from Papaver rhoeas and potential valorization of these extracts in dyeing applications

**DOI:** 10.3906/kim-2105-44

**Published:** 2021-10-19

**Authors:** Mohand Ouidir BOUSSOUM, Abdelkader ALI-NEHARI, Rachida OULDMOKHTAR, Beatrice GEORGE

**Affiliations:** 1 Faculty of Natural and Life Sciences, University Ibn Khaldoun, Tiaret Algeria; 2 Laboratory for Studies and Research on Wood Material, Lermab, Inra France

**Keywords:** *Papaver rhoeas*, dye, essential oil, mordant, fiber, valuation, dyeing

## Abstract

The aim of our work is to study the dyeing properties of extracts from natural sources such as *Papaver rhoeas* by modifying the conditions of the dyeing process, choosing other substrates that could be pretreated by innovative and environmentally friendly processes. The results obtained show a fairly high fixation of the extracts on the natural and artificial fibers. The use of mordants allowed the dye to be better fixed on the dyed fibers and to give several shades to the fabric. The rate of essential oil contained in *Papaver rhoeas* was considered important by the Clevenger process. Screening of extractive revealed the presence of polyphenols, saponins, sterols, alkaloids and flavonoids. In this study, the extractive from *Papaver rhoeas* was investigated as textile dyestuff.

## 1. Introduction

Scientific advances in the field of the valorization of natural substances derived from plants (leaves, bark, sawdust) make it possible to envisage, in the long term, that green chemistry products, substitutable and competitive, replace those derived from fossil materials, in the fields of energy and fine chemistry. As these fossils are being depleted, researchers are turning to easily renewable sources such as the *Papaver rhoeas*.

Natural dyes, usually extracted from minerals, animals or plants were widely used to dye natural fibers (wool, silk, cotton) or artificial (polyamides, polyacrylic, polyester) until they were supplanted by synthetic dyes in the 2nd half of the nineteenth century [1]. The latter, derived from petrochemicals, have certain advantages (wide range of colors, availability, cost, etc.) but also notable disadvantages (toxicity vis-à-vis humans and the environment). Indeed, these substances that can end up in industrial effluents are generally more resistant to biodegradation and persist in the environment [2].

As a result, industry has attracted interest in natural dyeing of textiles due to their biodegradability, nontoxic nature, nonallergic nature as well as environmental compatibility. Natural dyes include dyes obtained from animal, plant or mineral material without any chemical treatment, in such a way as to increase awareness of environmental and health risks. The most important source of natural dyes is plants, which, according to Merdan et al. [3], are usually obtained from leaves, flowers, cones, stem bark and root.

These natural substances can be extracted via traditional processes such as solvent extraction (most often water, with or without the addition of acid or base) [4], but ultrasonic processes, using supercritical fluids or enzymes can also be implemented.

In the process of dyeing from natural dyes, etching is very often used. It consists of a pretreatment of the fiber with a metal salt (alum and iron sulfate II mainly) to improve the fixation of the dye and which also has the effect of expanding the color palette [5]. Conventional mordants tend to be replaced also by natural mordants based on tannin-rich plants [6] or hypermetal accumulating plants [7].

Studying the dyeing properties of extracts from other sources such as *Papaver rhoeas *as proposed is therefore quite possible. Moreover, after a quick overview of the literature, it turns out that very little research has been conducted on this type of substances for this particular area of application [8,9], while the study of the antioxidant character of *Papaver rhoeas* has been the subject of several articles [10–12]. The few studies interested in the dyeing aspect show that it is possible to dye raw or cationized cotton (in the presence or absence of mordant) or silk (conventional dyeing process or under ultrasound) and that the substrates dyed have generally good resistance to washing, resistance to abrasion in dry, wet as well as a sweat fastness, just suitable, but a rather poor resistance to light.

We could enrich these first works by changing the conditions of the dyeing process, by choosing other substrates that would be possible to pretreat by innovative and environmentally friendly processes (surface corona treatment, under UV radiation, use of more green mordants, etc.).

## 2. Materials and methods

### 2.1.Textile materials

The different types of fabrics used when performing our experiments are shown in Table 1.

**Table 1:** Properties of textile materials used.

	Weight (g/cm2)	Thickness (mm)
Cotton	0.045	1
Wool	0.200	3
Viscose	0.038	1

In general, a cotton fiber can be broken down into six parts: cuticle, primary wall, winding layer, secondary wall, lumen wall and lumen. Different layers possess different chemical compositions and microstructures. Viscose is made from cellulose, a constituent of all land growing plant life. However, wool is a heterogeneous fiber, and the composition of the fiber is highly variable within as well as between sheep. These fibers are soft, smooth, cool, comfortable, and highly absorbent, but they do not insulate body heat, making them ideal for use in hot and humid climates.

### 2.2. Dye extraction

The extraction was done with a fiber-liquid ratio (F/L) of 1/10 (m/v).

The solvent used for the extraction of dyes is distilled water, on the one hand, and an aqueous solution of soda (NaOH) in different concentrations: 0.05 M; 0.1 M; 0.2 M; 0.3 M; 0.4 M and 0.5 M, on the other hand. A total of 15 g of Papaver are fed into an Erlenmeyer flask and covered with 150 mL of solvent, the whole being tightly closed with a layer of parafilm and aluminum foil on the neck. The set is brought to reflux for an hour under stirring. Without cooling step, the mixture is filtered under vacuum and the recovered filtrate will serve as the coloring material of the selected fibers with or without mordant. The extractable rate is determined after drying in the oven at 103 °C for 24 h of the residue obtained after extraction by the formula below:

(1)TE(%)=m1-m2m1x100

where 

m1: dry Papaver mass;

m2: Papaver mass after extraction and drying in the oven for 24 h at 103 °C.

### 2.3. Dyeing method

#### 2.3.1. Without mordant

The coloring solution is prepared by dilution to the fifth of the parent solution (solution obtained after extraction of Papaver). A total of 1 g of coloring fiber is fed into a 75 mL beaker and covered with 25 mL of coloring solution. The assembly being hermetically closed and put in a thermostated bath at 25 °C under stirring for 30 min to allow the adsorption of the dye by the fiber. After this adsorption period, the temperature is raised to 60 °C for 2 h to allow the dye to be fixed on the fiber. After this time, the colored fiber is removed and cleared of the remaining solution, then washed with 75 mL of distilled water at 60 °C for 30 min to rid the fiber of the remaining unfixed dye.

#### 2.3.2. With mordant

Before introducing the coloring solution, the fibers are initially treated with 25 mL of mordant based on:

Iron sulphate (FeSO4, 7H2O) to 1%;

Aluminium sulphate (Al2 (SO4)3) to 3%;

Acetic acid (CH3COOH) at 3%.

The fibers are soaked in 25 mL of mordant for 30 min at room temperature, after this treatment time, 10 mL of the prepared coloring solution is added to it.

### 2.4. Washing method

It is carried out according to ISO 105-C10 [13]. We prepared a washing solution using the Test detergent produced by SARL Hayat DHC Algeria licensed by Hayat Kimya Sanayi A.Ş. (İstanbul, Turkey), (5mL per 1L of water) the colored samples are washed in this soap solution at 60 °C for 30 min, with a material/liquid ratio of 1:50 (m/v). After washing, the material is rinsed with distilled water and dried at 70 °C for 48 h.

### 2.5. Color measurement

Color variation measurements are performed using a Datacolor D65°10 that provides the values of L*, a*, b*, a*, and b* representing the hue deviation from a reference.

### 2.6. Batch extraction

The sample of the plant material obtained by grinding, previously weighed, is brought into contact with the solvent mixture (ethanol-water, 80%) in the flask. The mixture is stirred and kept at a set temperature, depending on the operating conditions.

After a well-determined extraction time, the solid and liquid phases are separated by centrifugation at a speed of 3500 rpm for 20 min. The liquid phase containing the phenolic compounds is recovered and kept away from light [14].

Analysis using gas chromatography coupled to mass spectrometry (GC/MS).

Gas chromatography analysis coupled with mass spectrometry (GC/MS)GC-MS analysis is performed using a Perkin Elmer Clarus 680 gas chromatograph, coupled with a Perkin Elmer Clarus SQ8 mass spectrometer, a set driven by TurboMass v. 6.1 software and having a 2011 NIST MS Search 2.0 database.

Before injection, the samples are derived to facilitate the detection of all compounds present. For this, 2 mg of extractables are solubilized with 50 µL of bstfa + TMSCl 1% (a silylation agent) in a 2 mL pillbox. This preparation is placed for 120 min, closed pillbox, in an oven at 70 °C, to allow the reaction of the derivation agent on the extractables. The pillbox is then opened to allow the BSTFA to evaporate. A total of 1 mL of ethyl acetate is finally added to the pillbox to solubilize the silylated extractables, then 1 µL of the latter solution is injected splitless into the GC, via an injector heated to 250 °C.

Chromatographic separation is carried out with a DB-5ms stationary phase (dimethyl-/ diphenyl-polysiloxane 95%/5%; length: 30 m; internal diameter: 0.25 mm; film thickness: 0.25 µm) with a 40 min furnace temperature program, comprising a 2 min bearing at 80 °C, a rise to 10 °C min^–1^ up to 190 °C, a rise to 15 °C min^–1^ up to 280 °C, then maintained temperature for 10 min and finally a last step at 300 °C reached at 10 °C min^–1^ and maintained 14 min and a mobile phase consisting of helium at 1 mL/min.

After separation, the compounds are sent to the mass spectrometer via a transfer line thermostated at 250 °C and then ionized at 70 ev.

## 3. Results and discussion

### 3.1. Extractable rates

The extractable was used to dye different materials. While the solids were recovered after filtration and dried in an oven at 103 °C for 24 h and then weighed using a Kern ALS 12C-4n brand scale of 10–4 accuracy for use in yield calculations. The experiments were carried out in triplicate to ensure reproducibility of the results. The average values and standard deviations are shown in Table 2.

**Table 2:** Extractable rate (average of three tests, standard deviation) obtained.

Extractable rates (%)		
Extraction solvents	Water	9.667 ± 0.20
	Hot water	9.675 ± 0.06
	NaOH 0.05 M	10.22 ± 0.28
	NaOH 0.1 M	9.810 ± 0.50
	NaOH 0.2 M	10.24 ± 0.34
	NaOH 0.3 M	9.750 ± 0.20
	NaOH 0.4 M	9.940 ± 0.36
	NaOH 0.5 M	9.880 ± 0.17

It appears that the extractables rate varies from one solvent to another. Extraction rates give virtually the same result regardless of the type of solvent used. It is noted that the highest extractable rate is obtained with soda at a concentration of 0.2 M (10.24%) and the lowest is obtained for water extraction solvent (9.667%).

In the continuity of the valuation of extractables, it was discussed whether it was possible that these extractables were used to dye the fibers.

### 3.2.Dyeing of fibers without the use of a mordant

The results of fixing the dye obtained after extraction on natural and artificial fibers without the use of a mordant are shown in Table 3.

**Table 3:** Natural and artificial fiber coloring results without mordant.

		Tested supports		
		Cotton	Wool	Viscose
Extraction solvents	Water	+	–	–
	Hot water	+	+	+
	NaOH 0.05 M	+	+	–
	NaOH 0.1 M	–	+	–
	NaOH 0.2 M	–	+	–
	NaOH 0.3 M	–	–	–
	NaOH 0.4 M	–	–	–
	NaOH 0.5 M	–	–	–

Coloration: (+) presence, (–) absence.

From this table, it appears that the coloring of the three fibers used, namely cotton, wool and viscose was produced with hot water and was absent in the case of soda solutions at concentrations, respectively, of 0.3, 0.4 and 0.5 M and in the wool with soda at concentrations of 0.05, 0.1 and 0.2 M and with water, while the coloring of viscose was not produced by the other solvents.

The dye extracted using hot water gave gray shades on all the tinted fibers compared to the control fibers, unlike extracts obtained from aqueous solutions of soda (NaOH) or water, which do not have a great effect on the fibers. This can be explained by the affinity of the solvents used in relation to the composition of the fibers used.

Having obtained better results with extractables without mordant, we thought to make coloring using mordants iron, aluminum and acetic acid to better fix the dye on the fiber or to make shades of colors.

### 3.3.Influence of mordants 

The results of fixing the dye obtained after extraction on natural and artificial fibers with the use of a mordant (iron, aluminum and acetic acid) are presented in Table 4.

**Table 4:** Staining results of the fibers used.

Mordants		FeSO4, 7H2O (1%)			Al2 (SO4)3 (3%)			CH3COOH (3%)		
Tested supports		Cotton	Wool	Viscose	Cotton	Wool	Viscose	Cotton	Wool	Viscose
Extraction solvents	Water	+++	+++	++	–	–	–	+	+	+
	Hot water	+++	+++	+++	+	+	+	++	++	+
	NaOH 0.05 M	++	+++	++	–	–	–	++	+	+
	NaOH 0.1 M	++	++	+	–	–	–	+	+	–
	NaOH 0.2 M	+	+	–	–	–	–	+	+	–
	NaOH 0.3 M	+	+	–	–	–	–	+	+	–
	NaOH 0.4 M	+	+	–	–	–	–	+	–	–
	NaOH 0.5 M	-	+	–	–	–	–	–	–	–

According to the results, there is a strong fixation of the extracts obtained with water, hot water and aqueous solutions at low concentrations of sodium hydroxide of the various samples initially treated with iron sulfate (FeSO_4_, 7H_2_O). This means that the fiber has become darker than the standard. A weak fixation for fabrics tinted with aqueous solutions of NaOH from 0.2 to 0.4 M for natural fibers was observed along with a complete absence of staining for artificial fiber. The tint remained unchanged in color, which leads us to the conclusion that the mordant did not have a great effect for the color shade. In addition, medium fixation of the extracts was obtained on natural fibers initially treated with acetic acid (CH_3_COOH), and low to a total absence of staining on the artificial fiber. Therefore, we found no staining of the three fibers treated with aluminum sulfate (Al_2_ (SO4)_3_) of all the coloring extracts, except for the extract obtained with hot water which gave a slight coloring to the fiber.

**Figure 1:**
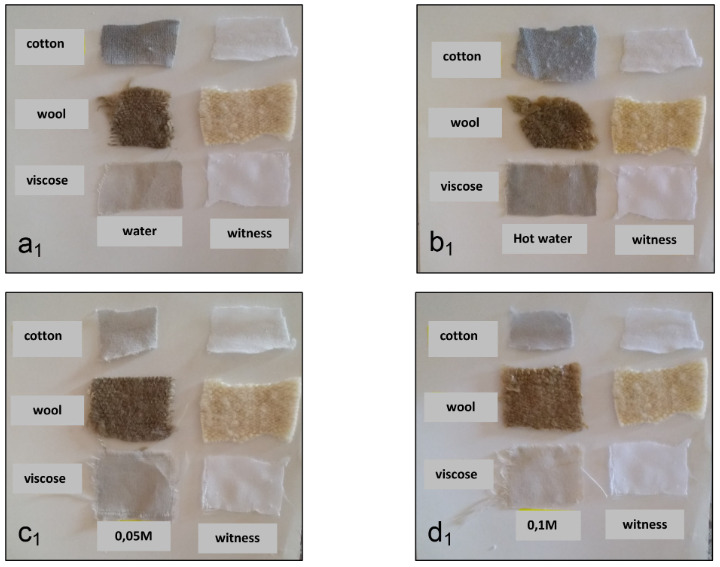
Color obtained after coloring of samples treated with iron sulphate. a1: dyeing with dye extracted by water. b1: dyeing with dye extracted by hot water. c1: dyeing with dye extracted by NaOH 0.05 M. d1: dyeing with dye extracted by NaOH 0.1 M.

**Figure 2:**
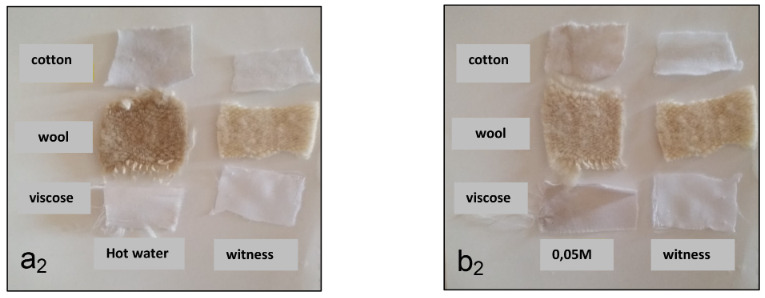
Color obtained after staining samples treated with acetic acid. a2: dyeing with dye extracted by hot water. b2: dyeing with dye extracted by NaOH 0.05 M.

Fabrics dyed with extracts of the *Papaver rhoeas *with the use of a mordant gave a better coloring, and these are shown in Figures 1 and 2 (left: dyed fabrics, right: control fabrics).

From these figures it emerges that the mordants used in combination in different ratios gave different shades to the naturally and artificially dyed fabric samples. Color intensity results and different shades depend on the mordant used.

Therefore, the best staining results were obtained after treatment of the fibers with iron sulfate (FeSO_4_, 7h2o). The fibers appear to take a dark coloration compared to the standard when using the mordant at the concentration of 1% on the tissues. These results show a grey shade of color on all tinted fabrics. Beside, a low fixation was determined for fabrics tinted by aqueous solutions with high concentrations of NaOH.

In addition, there is a slight grey coloring for natural fibers treated initially by acetic acid 3% with aqueous and alkaline extracts, unlike artificial fibers where there was a slight fixation only with aqueous extracts.

In contrast, the dyes obtained using 3% aluminum were the lowest of all samples. The use of aluminum mordant had no effect on the fibers with alkaline extracts, however, it can be noted that the use of aluminum mordant had a slight effect compared to the standards on natural and artificial fibers with extracts obtained in hot water.

This, once again, confirms that aqueous extracts dye the fibers better than alkaline extracts.

### 3.4. Color fastness on fiber

**Figure 3:**
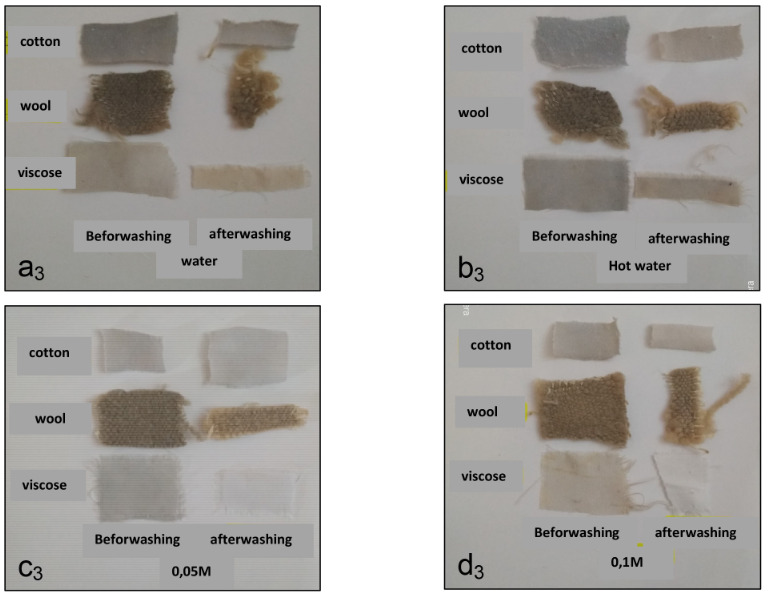
Dyed with aqueous Papaver extracts treated with iron sulfate and leached. a3: dyed with aqueous Papaver extracts and leaching-water. b3: dyed with Papaver aqueous extracts and leached-hot water. c3: dyed with aqueous extracts of Papaver and leaching-NaOH 0.05 M. d3: dyed with aqueous extracts of Papaver and leaching-NaOH 0.1 M.

**Figure 4:**
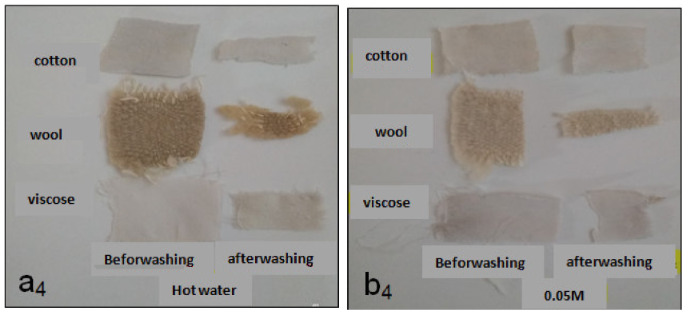
Dyed with aqueous Papaver extracts treated with acetic acid and leaching. a4: dyed with aqueous Papaver extracts and leaching-hot water. b4: dyed with aqueous extracts of Papaver and leaching-NaOH 0.05 M.

After this etching step, the colored fibers were leached with soapy water (Figures 3 and 4). The use of the mordant leads to a better resistance of the dyes to leaching.

According to the results obtained after leaching (Figures 3 and 4), the coloring of the fibers was not strongly influenced by washing, and regardless the nature of the extract (aqueous or alkaline), the colors remain linked to the textile support. In addition, regardless of the chemical nature of the fiber (natural or artificial) leaching has little influence on the rigidity of the holding of the dye by the fiber. So, the textile backing color bond is so strong enough in general.

In addition, the color fastness for friction gave excellent results in both dry and wet friction. Wool dyed fabrics gave better resistance to washing.

### 3.5. Analysis by (GC/MS)

Table 5 shows the results of chemical analysis by gas chromatography coupled with mass spectrometry (GC-MS) of different extractables of the *Papaver rhoeas*, which aims to separate different volatile and semivolatile compounds from a mixture and analyze them qualitatively, semiquantitatively and quantitatively.

**Table 5:** Compounds identified in Papaver rhoeas extractables.

Solvent	Retention time (min)	Identified compounds	Formula	Relative composition (%)
Water	4.372	Silane, ethyl	C2H8Si	0.189%
	6.635	4H-Pyran-4-one, 2,3-dihydro-3,5-dihydroxy-6-methyl	C6H8O4	/
		2,3-Dihydro-3,5-dihydroxy-6-methyl-4H-pyran-4-one		
	8.229	5H-Cyclopropa[3,4]benz[1,2-e]azulen-5-one, 3,9,9a-tris(acetyloxy)-3-[(acetyloxy)methyl]-2-chloro-1,1a,1b,2,3,4,4a,7a,7b,8,9,9a-dodecahydro-4a,7b-dihydroxy-1,1,6,8-tetramethyl-, [1a(1aα,1bβ,2α,3β,4aβ,7aα,7bα,8α,9β,9aα)]-	C28H37ClO11	0.486%
	8.851	1,3,5-Triazine, 2-(propylthio)-4,6-bis(trichloromethyl)	C8H7Cl6N3S	0.083%
	8.894	Chromium, tricarbonyl[(η5-2,4-cyclopentadien-1-yl)cobalt][μ-(η2:η6-4,5-diethyl-2,2,3-trimethyl-1-phenyl-1-aza-2-sila-5-boracyclopent-3-ene-B5,N1)	C23H29BCoCrNO3Si	0.148%
	16.060	Hematoporphyrin	C34H38N4O6	0.394%
	16.093	2β,4a-Epoxymethylphenanthrene-7-methanol, 1,1-dimethyl-2-methoxy-8-(1,3-dithiin-2-ylidene)methyl-1,2,3,4,4a,4b,5,6,7,8,8a,9-dodecahydro-, acetate	C27H38O4S2	0.240%
	16.105	n-Hexadecanoic acid	C16H32O2	/
		1-(+)-Ascorbic acid 2,6-dihexadecanoate	C38H68O8	/
		Hexadecanoic acid	C16H32O2	/
	16.183	Hematoporphyrin	C34H38N4O6	0.293%
	16.390	Hexadecanoic acid, ethyl ester	C18H36O2	/
	16.696	2,3-Dicarbaheptaborane(7), 2,3-dimethyl-	C4H11B5	2.596%
	16.869	Dinaphto[2,3-b:2,3-n]perylene	C36H20	0.134%
	18.443	Ethyl cyanoacetate	C5H7NO2	0.066%
	18.480	Hexacosane	C26H54	/
		Tetracosane	C24H50	/
	18.495	Pentacosane	C25H52	/
	20.071	Pentacosanoic acid, 17,18-bis(methylthio)-, methyl ester	C28H56O2S2	0.083%
	20.765	.beta.-Tocopherol	C28H48O2	/
		®-6-Methoxy-2,8-dimethyl-2-((4R,8R)-4,8,12-trimethyltridecyl)chroman		
		.delta.-Tocopherol, O-methyl-		
	21.095	Hexatriacontane	C36H74	/
		Dotriacontane	C32H66	/
		Tetratriacontante	C34H70	/
	30.497	Diethyl Phthalate	C12H14O4	93.693%
	34.268	Estra-1,3,5(10)-trien-17β-ol	C18H24O	1.595%
Water-ethanol	5.658	Silanediol, dimethyl-	C2H8Si	2.034%
	10.706	Oxime-, methoxy-phenyl-	C8H9NO2	13.668%
	19.746	Cyclotrisiloxane, hexamethyl	C6H18O3Si3	3.373%
	32.645	Pentaethylene glycol	C16H36O6Si	22.128%
	35.242	Hexaethylene glycol, TBDMS derivative	C18H40O7Si	36.935%
	39.128	(2S,2’S)-2,2’-Bis[1,4,7,10,13-pentaoxacyclopentadecane]	C20H38O10	8.896%
	42.034	(1S,14S)-Bicyclo[12.10.0]-3,6,9,12,15,18,21,24-octaoxatetracosane	C16H30O8	12.966%

Diethyl phthalate has been found in large quantities in water extracts, as well as other trace compounds such as hematoporphyrin, ethyl cyanoacetate, 2,3-dicarbaheptaborane (7), 2,3-dimethyl-and estra-1,3,5(10)-trien-17β-ol. While the main compound in ethanol extracts is hexaethylene glycol, tbdms derivative. The ethanol extract contains in particular oxime-, methoxy-phenyl-, pentaethylene glycol, as well as (1s,14s)-bicyclo [12.10.0]-3,6,9,12,15,18,21,24-octaoxatetracosane.

## 4. Conclusion

The present study revealed that the flower of the *Papaver rhoeas* has, on the one hand, the dyeing potential as a natural source for dyeing fibers (cotton, wool and viscose), and, on the other hand, is a source rich in essential oils. Thus, there is a possibility of the valorization of its extracts in the fields of antibacterial, antioxidant or antifungal. The use of the aqueous and alkaline extractables of *Papaver rhoeas* as a source of natural dye for dyeing natural and artificial fibers has yielded excellent results. Different grey shades were noticed on the tinted fibers. Experience has shown that the tint with the extracts obtained in hot water is better than that obtained through water and sodium hydroxide solutions. The use of mordants made it possible to better fix the dye on natural and artificial fibers and give several variations of shades. The best dyeing results were obtained by the use of iron-based mordant. The best shade was obtained with natural fibers (cotton, wool). The dye of the fibers tinted by the extracts of the *Papaver rhoeas* showed satisfactory resistance to washing and rubbing.

Photochemical screening revealed the presence of different groups of molecules such as polyphenols, saponins, sterols and alkaloids as well as flavonoids in extractables.

In general, the valorization of the extractables of the *Papaver rhoeas* gives a great added value to the exploitation of this plant.
